# Exploration of a co-care service for chronic care management in patients with hypertension: A participatory qualitative study (Phase 1)

**DOI:** 10.1016/j.rcsop.2026.100842

**Published:** 2026-09-13

**Authors:** Fanny Mulder, Marina Lopes Cardoso, Alessia Romer, Kristie Rebecca Weir, Alice Panchaud, Stephen Philip Jenkinson, Karen Alexandra Maes

**Affiliations:** a Institute of Primary Health Care (BIHAM), University of Bern, Bern, Switzerland; b Graduate School for Health Sciences (GHS), University of Bern, Bern, Switzerland; c Swiss Pharmacists Association pharmaSuisse, Liebefeld, Switzerland; d Sydney School of Public Health, Faculty of Medicine and Health, University of Sydney, Sydney, Australia; e Service of Pharmacy, Lausanne University Hospital and University of Lausanne, Lausanne, Switzerland

**Keywords:** Integrated care, Interprofessional care, Primary care, Hypertension, Chronic disease management, Qualitative research

## Abstract

**Introduction:**

In Switzerland, more than 80% of diagnosed individuals with hypertension are prescribed at least one blood pressure-lowering agent, yet 60% have uncontrolled blood pressure. To address this burden, co-care has been shown to improve systolic blood pressure control and to be cost-effective. Currently, no co-care service for hypertension management involving pharmacists, general practitioners (GPs), and patients is available in Switzerland.

**Aim:**

This qualitative study aimed to identify key elements supporting the future development of a co-care service for chronic hypertension management, informed by healthcare professionals' (HCPs) and patients' perspectives and experiences.

**Methods:**

This participatory study included semi-structured interviews with six pharmacists and six GPs, and a focus group of six patients with hypertension. Recruitment targeted diverse Swiss regions and practice settings to capture multiple perspectives and help shape the co-care service. Transcripts were coded using thematic analysis.

**Results:**

Pharmacists, GPs, and patients identified effective communication, mutual trust, and clear role differentiation as key elements to support the future development of a co-care service. Structured collaboration tools, such as shared hypertension guidelines, were viewed as necessary to support interprofessional collaboration. Improved information sharing, potentially through an electronic health record, was seen as key to care coordination, although concerns about data security remained. Contextual barriers, including political factors, highlighted the need for local implementation strategies.

**Conclusion:**

The key elements of a future co-care service for chronic hypertension management depend on mutual trust, a local and shared way of communication and collaboration among HCPs and patients, combined with a holistic patient-centered approach.

## Highlights

• Effective communication and mutual trust.
• Shared clinical guidelines and clear role differentiation.
• A flexible framework to support implementation in different practice settings.
• A dedicated patient contact person for coordinated care and information support.

## 1 Introduction

Cardiovascular diseases (CVDs) remain the leading cause of death globally.^1^ In 2023, high systolic blood pressure contributes to an estimated 10.5 million CVD deaths accounting for over half of CVD burden worldwide,^2^ many of which were preventable through hypertension treatment.^1^ In Switzerland, hypertension affects a substantial portion of the population, with 22% of men and 17% of women either diagnosed with hypertension or receiving hypertension treatment in 2022.^3^ Although more than 80% of diagnosed individuals are prescribed at least one blood pressure–lowering agent, approximately 60% still have uncontrolled blood pressure.^4^ Effective hypertension care requires long-term self-management and adherence to treatment. Moreover, adherence to antihypertensive medications is associated with reductions in preventable hospitalizations.^5^ Nearly 75% of all Swiss hospitalizations due to hypertension are considered potentially avoidable, with an estimated annual cost of over CHF 16 million (about EUR 17.5 million in June 2026).^6^ To reduce this burden, co-care has been introduced, and is defined as an approach that emphasizes the complementary role of healthcare professionals (HCPs) in supporting individuals' own resources for managing their health and the use of appropriate tools to support knowledge sharing among all actors.^7^ It has been shown to improve long-term systolic blood pressure control^8^ and is based on by evidence demonstrating its cost-effectiveness in hypertension management.^1^

In this context, pharmacists can play a crucial role in a co-care service due to their accessibility and expertise in medication management. Co-care is defined as a partnership in care where HCPs and patients collaborate to provide support in managing patients' own health and the use of appropriate tools to support knowledge sharing among all actors.^7^ A systematic review found that pharmacist-led prescribing services (non-medication initiation, continuation, and modification of medication) were associated with easier access to care, shorter waiting times, and more convenient opening hours compared to general practitioners' (GPs) practices.^9^ Co-care services for hypertension management, involving pharmacists, have been implemented in several countries and shown to improve blood pressure outcomes. In Germany, a traffic light–based referral protocol used by pharmacists enabled the detection of both patients with undiagnosed hypertension and uncontrolled blood pressure among patients with diagnosed hypertension.^10^ Most pharmacists expressed a willingness to continue using the referral guidelines, which were perceived as more useful than the standard offer. In Canada, a randomized controlled trial showed a significant reduction in CVD events when pharmacist-led medication therapy management was added to usual care.^11^

In Switzerland, several services in the field of hypertension management are used in routine pharmacy practice. For instance, blood pressure measurement services are available to patients in community pharmacies.^12^ Additionally, a pharmacy-based cardiovascular risk assessment service (Heart Check, in German: HerzCheck®) provided by the Swiss Heart Foundation allows pharmacists to measure blood lipids, blood sugar, blood pressure, waist circumference, and assess CVD risk factors.^13^ An evaluation of this pharmacy-based cardiovascular risk assessment service has shown a positive influence on patients' health behaviors and lifestyle management.^13^ In the Swiss French-speaking region, a co-care service involved GP practice nurses, who handled blood pressure and lifestyle counseling, and pharmacists, who focused on medication adherence.^8^ As a result of this co-care, daytime systolic blood pressure was significantly reduced after 12 months, although diastolic changes were not significant, and the overall impact remained limited due to local implementation (i.e., selected outpatient clinics) and lack of remuneration. Further development of co-care services is needed in Switzerland to prevent gaps in continuity of care.

A standardized co-care service involving pharmacists, GPs, as well as patients and/or their relatives, has demonstrated benefits for patient care in Switzerland and internationally. As with any new service, it needs to reflect real-world practice, HCP and patient needs, and context, thereby improving feasibility, acceptability, and sustainability. Thus, stakeholder involvement (i.e., HCPs and patients) is essential from the initial stages of development to ensure its success. Building on this principle, a structured and context-adapted co-care service is required to implement such collaboration in routine practice. Accordingly, the present participatory qualitative study aimed to identify key elements that may support the future development of a co-care service for chronic hypertension management, informed by the perspectives and experiences of HCPs and patients in primary care.

## 2 Methods

### 2.1 Design and setting

This qualitative study followed a participatory approach to explore the experiences of pharmacists, GPs, and patients with hypertension regarding hypertension management within Swiss primary care settings, as well as their perceptions of a new co-care service. Focus groups were used for patients to efficiently capture diverse perspectives and to facilitate interactive discussions that elaborated on individual contributions.^14^ Individual semi-structured interviews were conducted with pharmacists and GPs due to scheduling constraints. The study was reported according to the consolidated criteria for reporting qualitative research (COREQ) guidelines (see Appendix A – COREQ (COnsolidated criteria for REporting Qualitative research) guidelines).^15^ Ethics approval was waived by the Ethics Committee of the Canton of Bern, Switzerland, for the focus group, since it does not fall under the purview of the Swiss Human Research Act (BASEC-Nr-2023-01415). This study constituted the first step (Phase 1) in the development of a new co-care service intended for integration into Swiss primary care settings.

### 2.2 Interviews with pharmacists and GPs

The research team invited pharmacists and GPs, who were either currently working or undergoing specialization within the Swiss primary care setting. Participants were invited for an individual semi-structured interview via email using convenience and purposive sampling. Participating pharmacists were recruited through the Swiss Pharmacists Association pharmaSuisse, while GPs were recruited through the network of the Institute of Primary Care, University of Bern. Pharmacists and GPs were selected from different regions of Switzerland, varying in geographical setting (urban, suburban, and rural), linguistic context (German, French, and Italian), and medication dispensation regulations, in order to capture regional diversity.^16^ Switzerland is divided into 26 constituent states, known as cantons, each with its own regional government and a high degree of political and administrative autonomy within the Swiss federal system. In addition to pharmacy-based medication dispensation, some cantons allow GPs to dispense medication directly in their practice (i.e., self-dispensation), whereas others permit this only under specific conditions, such as the number and proximity of nearby pharmacies (i.e., mixed dispensation).

The interview topic guide was developed based on a literature review with a focus on the current management of hypertension in pharmacies and GP practices, participants' views on how communication should occur, and their vision for a new hypothetical co-care service for managing hypertension (see Appendix B – HCPs' interview topic guide). No predefined co-care model was presented, instead, participants were invited to engage in open, exploratory discussions. To ensure methodological rigor and relevance, the guide was developed by MC, a pharmacy master's student, and refined with collaborative input from the research team. It was reviewed by FM, a pharmacist and PhD student, as well as KAM and SPJ, senior pharmacists with clinical and research expertise.

After an open discussion about a future co-care service, the existing German referral traffic light guidelines were presented to HCPs in the last part of the interview guide.^10^ These guidelines allow pharmacists to classify patients, with or without a hypertension diagnosis, into three risk categories based on the average of three blood pressure measurements taken after a five-minute rest. Red indicates the need for a GP evaluation within the next 4 weeks, yellow indicates that measured values should be communicated to the GP at the next regular visit, and green indicates well-controlled blood pressure requiring regular monitoring. These guidelines were selected because they are currently used in the German healthcare system and represent a practical tool^17^ with potential applicability within the co-care service. Their feasibility and implementation within the co-care service were explored.

A single pilot interview with a pharmacist led to minor adaptations of the interview topic guide, and its data was excluded from the analysis. The semi-structured interviews with pharmacists and GPs were conducted and recorded online via the platform Zoom® in April 2024. The interviews lasted between 28 and 50 min with a median interview duration of 34 min and 37 s. Written informed consent was signed from the pharmacists and GPs before their interview. No prior relationship was established between researchers and participants, and HCPs had no access to the interview questions or study aims, apart from the information in the invitation letter (see Appendix C – Invitation letter for a qualitative interview). MC, a novice interviewer, conducted all interviews, with supervision provided by FM or SPJ during the first four interviews to ensure methodological rigor before MC proceeded independently.

### 2.3 Focus group of patients

For the patients' focus group, a convenience sample of six patients were recruited from consumer groups including the Diabetes Club, the Heart Foundation Affected Council, and Diabetes Switzerland, and a recruitment agency. Participants were eligible if they were aged 18 years or older, self-reported a diagnosis of hypertension, and lived in the German-speaking region of Switzerland. Participants received compensation for travel costs and CHF 150 (about EUR 160 in June 2026) for their participation. Participants consented verbally to audio recording at the start of the focus group.

The focus group integrated three thematic questions about co-care services for hypertension, based on the same literature review as the HCPs interview guide and development pathway, and no pilot was conducted prior to data collection. We recognized that patients would not understand the concept of a co-care service, so we asked questions about their views on interprofessional services, including pharmacists and GPs, for hypertension management. Patients discussed open-ended questions regarding their experiences with hypertension management, communication between pharmacists and GPs, and their views on potential pharmacy-based services for hypertension care, such as blood pressure monitoring in pharmacies (see Appendix D - Patient interview topic guide).

The focus group was led in person in March 2024 and took place in an external office in Bern, Switzerland, and was recorded using two independent audio recorders placed at opposite sides of the table to ensure comprehensive and safe audio capture. No pilot was conducted prior to the focus group. It was moderated by JL, with a background in communication science and sociology. AR, a pharmacist and PhD student and AS, with qualifications in psychological and social sciences, served both as co-facilitators and workshop observers. MC also observed the focus group. The focus group lasted 54 min.

### 2.4 Data analysis

The interviews and focus group were transcribed using the transcription feature of Microsoft Word® (Windows 11, version 2402). MC verified the accuracy of the interview transcriptions, while AR verified the focus group transcription, both by listening to the audio recordings. No transcripts were returned to participants for review. The sample size was considered sufficient for this exploratory qualitative study, as it included all three key actors' groups (patients, pharmacists and GPs) of the service and allowed a relevant variation sampling and a rich and in-depth data analysis to identify recurrent themes appropriate to the study aim. All transcripts were analyzed using MAXQDA® software (version 24.2.0). Three investigators (MC, AR, FM) conducted the step-by-step process of thematic analysis by Naeem et al.,^18^ using an inductive approach with themes derived from the data. MC conducted the analysis of all interviews and the focus group transcripts. AR independently analyzed the six German-language interviews, FM the French-language interviews, and both additionally contributed to the focus group's transcript analysis.

An inductive approach was used to develop individual keywords, codes, and themes. The development of codes and themes was compared and discussed among the three researchers. A researcher (FM) conducted the analysis a second time to ensure accuracy and consistency of the findings. Interviews and focus group data were analyzed in their original language, and the quotes in the results section were translated using a back-translation process. Translation adaptations were repeated until equivalence was ensured.

The qualitative study was conducted by a research team, with the analysis primarily performed by pharmacists (FM, MC, AR, SPJ, KAM) engaged in research promoting interprofessional collaboration and the pharmacist's role. KW, an experienced qualitative researcher, supported the structuring of the analysis and critically reviewed the interpretation of participants' perspectives.

## 3 Results

### 3.1 Demographics

The HCPs interviewed included six pharmacists and six GPs (Table 1), with an even distribution of German- and French-speaking interviews. The sample included HCPs with under 1 year to 31 years of experience, with a median of 11.3 years. Most HCPs worked in regions where only pharmacists dispense medication, but other medication-dispensing models were also covered.

**Table 1:** Demographics of the participating healthcare professionals (pharmacists *n* = 6, general practitioners *n* = 6).

Participant identification^a^	Profession^a^	Age	Year of experience	Working location	Working region (Canton)^b^	Type of medication dispensing^c^	Language of interview
Ph1	Pharmacist	39	15	Urban	Vaud	RX	French
Ph3	Pharmacist	39	15	Urban	Basel City	RX	German
Ph4	Pharmacist	39	15	Rural	Solothurn	SD	German
Ph5	Pharmacist	26	2.5	Suburban	Bern	MD	German
Ph7	Pharmacist	37	12.5	Urban	Fribourg	RX	French
Ph8	Pharmacist	39	10	Urban	Ticino	RX	French
GP2	GP	47	12	Rural	Bern	MD	German
GP6	GP	36	5	Suburban	Bern	MD	German
GP9	GP	57	31	Rural	Solothurn	SD	German
GP10	GP	38	7	Suburban	Fribourg	RX	French
GP11	GP	26	0.5	Rural	Vaud	RX	French
GP12	GP	46	17	Urban	Zürich	SD	German

a Participant Identification and profession: Pharmacist (Ph) and general practitioner (GP).

b Canton: A Swiss canton is one of Switzerland's 26 constituent states, each with its own regional government and a high degree of political and administrative autonomy within the Swiss federal system.

c Type of medication dispensing: In addition to pharmacy-based medication dispensation (RX), Swiss regions allow GPs to dispense medication directly in their practice (i.e., self-dispensation, SD), whereas others permit this only under specific conditions, such as the number and proximity of nearby pharmacies (i.e., mixed dispensation, MD).

Six patients participated in the focus group (Table 2). All patient participants were German-speaking, and gender was evenly distributed. All participants self-reported a diagnosis of hypertension, and three had diabetes. The median age of patients was 62 years.

**Table 2:** Demographics of the participating patients (*n* = 6).

Patients ID	Age	Gender	Language
P1	Not-reported	man	German
P2	Not-reported	man	German
P3	69	man	German
P4	55	woman	German
P5	70	woman	German
P6	52	woman	German

Abbreviation: P = Patient

### 3.2 HCPs' interviews

Pharmacists and GPs explored during the interviews their respective roles in the current management of hypertension in the primary care setting. Pharmacists' roles primarily focused on medication-related tasks (e.g., medication dispensation, adverse effects monitoring), whereas GPs' responsibilities focused on patients' hypertension management, including treatment decisions and prescription. Blood pressure monitoring can be conducted both in pharmacies and GP practices. Additionally, pharmacists and GPs discussed how they would envision a new co-care service for patients with hypertension. Four main themes emerged from the interviews with HCPs: 1) role differentiation and collaboration within the co-care service, 2) elements of the co-care service foundation, 3) beneficial aspects of a co-care service, and 4) implementation barriers to a co-care service for hypertension.

#### 3.2.1 Role differentiation and collaboration within the co-care service

To ensure successful collaboration within a new co-care service, participants emphasized the importance of mutual trust and understanding among HCPs. This could be achieved more easily when HCPs' workplaces were located nearby, and when clear definition of procedures, tasks, and roles were in place. This included a designated HCP taking responsibility for treatment coordination for hypertension (i.e., structured patient care coordination and information sharing among HCPs), with one participant noting that the GP would be best suited for this responsibility.

*«That there is an agreement between GPs and pharmacists, with a precise list of tasks. Who is responsible for what? (…) When does a patient belong to the GP, when does the GP want to see the patient, when does it fall within the scope of the pharmacist to determine when adjustments may be made? » (Ph5)*

Building on their current responsibilities, participants emphasized the possibility for pharmacists to perform therapeutic education and take a greater role in the achievement of patients' blood pressure targets in their daily practice. Additionally, most HCPs would be open to a new co-care service in which pharmacists would adjust medication dosage in consultation with the prescribing GPs, provided that pharmacists receive specific training and have increased access to patients' medical records.

*« It would be good if he/she (the patient) could visit the pharmacy from time to time to have his/her blood pressure measured. And then, also the monitoring, the compliance to taking medication.» (GP10)*

*«With our current training? Yes and no. It depends. (…) No. So basically, we could certainly do it, but we would need further training in this field.» (Ph3)*

HCPs recognized that patients contribute actively to their disease management, including taking prescribed medication, monitoring their blood pressure, and adopting recommended lifestyle measures. Patients were encouraged to share their needs and any challenges encountered with their treatment plan with their HCPs. One GP pointed out the decision-making process as a partnership with the patient.

«*Of course, they have to take their medication regularly. I think that's the be-all and end-all and then just monitor the therapy regularly or do it at home or in a pharmacy. That they are aware of these key points and take their therapy and try to implement lifestyle recommendations,* etc.» *(Ph3)*

#### 3.2.2 Elements of the co-care service foundation

In this theme pharmacists and GPs discussed that the co-care service requires clear protocols, tailored patient monitoring, lifestyle management, and robust interprofessional communication through shared electronic health record (EHRs). Participants shared their views on the existing German traffic light guidelines, a clear protocol used by pharmacists for hypertension follow-up that classifies patients into three risk categories based on blood pressure values and supports referral to GPs and could be adapted for use in Switzerland. HCPs often regarded the blood pressure thresholds as too strict, with some important aspects, such as adjustments for age, comorbidities, or patient symptoms, frequently overlooked. However, most pharmacists found the German traffic light guidelines important as a baseline reference and would welcome a similarly clear approach, with some adjustments, for the co-care service. Furthermore, integration of lifestyle management consultations (i.e., smoking cessation, blood lipid control, nutritional advice, weight reduction, and stress management) should be incorporated into such guidelines and were considered a key component of the co-care service.

«*I think that by strictly following this scale, we would produce unnecessary visits to the GP. So, I think the way the limits are now set in this system is very restrictive. (…) » (Ph4)*

Participants discussed how blood pressure measurement should be conducted and managed within the co-care service. Both pharmacists and GPs noted that blood pressure measurements should be tailored to individual patient needs and comorbidities, emphasizing that a single elevated value cannot define hypertension. A 24-h ambulatory blood pressure monitoring could be used in pharmacies to identify hypertension, which would then be confirmed by a GP diagnosis. All HCPs found that pharmacists were qualified enough to interpret blood pressure measurements when a dosing scheme was changed, and refer patients to GPs, when necessary. Participants mentioned that, to conduct a high-quality co-care service, pharmacists should have additional training and be responsible for the qualifications of their team. It was emphasized that a consultation room was necessary to ensure patient privacy.

Communication was seen as one of the most important aspects of the co-care service by participants to have all patients' information across different healthcare facilities. Mixed views were shared by HCPs on direct phone contact, with some considering it an efficient method for information exchange, while others questioned its reliability in reaching the intended HCP. The use of e-mails was often seen as positive. However, for most participants, the best way to communicate would be through an EHR. Participants were keen to discuss processes and modes of communication with closely collaborating HCPs, aiming to ensure effective interprofessional exchange and clarify local practices around their patients.

*« So there again, what is important is that groups working around a patient, or local networks of professionals, define their way of working.» (Ph1)*

*«In an ideal future, I think EHR would be a good thing. And it is true that there is an easy interface where you can have follow-up of vital sign parameters (…) » (GP11)*

However, even if patients have the possibility to receive more systematic care in pharmacies with the co-care service, most GPs emphasized the importance of patients continuing to come to their practice regularly, viewing it as central to their professional role and to providing high-quality care.

*«That the patient still comes to us from time to time. Simply because the whole relationship work is very important in general practice. And if everything is outsourced and we are really just the decision-makers without knowing the patients and seeing them regularly, then a major task of general practice will fall away.»* (*GP6*)

#### 3.2.3 Beneficial aspects of a co-care service

In general, HCPs perceived the co-care service as beneficial for patients' blood pressure management and education, while also improving access to HCPs, reducing GP workload, and fostering collaboration between pharmacists and GPs. Pharmacists and GPs highlighted that measuring blood pressure more systematically in pharmacies could improve target achievement and measurement accuracy. Moreover, the follow-up of patients could be improved by adding an HCP to the care pathway, and the ability to monitor patients who visit their GPs less frequently were mentioned.

*«They go anyway to the pharmacy at least every 3–4 months to get their medication. And if you can take a blood pressure reading, which is then sent to us, we already have a value, because people often come back a year later and say, ‘Oh, unfortunately I forgot to bring the blood pressure diary with me’ and then another year has passed, hasn't it?» (GP6)*

Participants stated that a co-care service could mean that patients see HCPs more often, who could contextualize blood pressure readings from both pharmacies and home measurements, providing more information about their medication. HCPs felt that with such improvement, medication adherence and patients' understanding of their disease and treatment might increase.

*«We simply know from family medicine that patients are bad at taking medication, that they forget to take it (…) But regarding the professional care they probably have too little input (…). And that would be important for the patient to simply find out more about themselves and their disease.» (GP12)*

All pharmacists and several GPs mentioned the convenience of pharmacy access, especially because there was no need for appointments or long waiting times.

*«I think it saves time for the patient, because they can do it relatively casually when getting their medication, for example. And then it takes maybe 10 or 15* *min in total, without having to plan the whole thing with an appointment and then the waiting room and then having to make another extra appointment.» (Ph4)*

With pharmacists taking more responsibility for patients' hypertension management (i.e., blood pressure follow-up with referral, when necessary), this co-care service was perceived to reduce the overall workload of GPs, a factor considered particularly relevant in the context of an increasing GP shortage. Additionally, a reduction in costs was also mentioned, due to task shifting to pharmacists in hypertension management.

*«(…) if it was installed and used, it would ease a lot of the burden on the GP. And we have a shortage of GPs, and that would be something that could be taken off their shoulders (…)» (GP9)*

*«Because of the structure or how the pharmacy is organized, I think it is also cheaper for the system. (…). » (Ph4)*

#### 3.2.4 Implementation barriers to a co-care service for hypertension

Key barriers to implementing the co-care service were resistance to changing current practices, limited HCP collaboration, and regulatory constraints. At present, HCPs mentioned insufficient trust between one another, due to lack of collaboration and communication. Enhancing interprofessional collaboration was perceived as potentially increasing the administrative burden associated with following patients across different healthcare facilities.

*«Not just a theoretical willingness, but a real and practical one. No longer working in separate silos as a profession but working together as a team toward a goal. We are a long way from that.» (Ph8)*

*«The obstacle is that there is no well-established method of communication to inform the GP of the results of this measurement.» (Ph1)*

Participants mentioned that both patients and HCPs were used to their actual care and that they were sometimes reluctant to change or involve other HCPs in their habitual practices.

*«The habit. Patients are used to having their blood pressure checked by their GPs. Also habits of us GPs. We just do it that way. Blood pressure feels like our job, but it should not be, so there needs to be a rethink, I think that is the main problem.» (GP9)*

Some participants highlighted the challenges posed by the political and regulatory context in certain regions of Switzerland, particularly in areas where GPs are allowed to dispense medications themselves (self-dispensation). This practice often led to limited collaboration between GPs and pharmacists.

*«That's why I am not so focused on collaboration with pharmacies, because it is not so strong here in this canton [Solothurn]. (…) I do not see the pharmacy of all places as the first partner here in this canton (…)»* (*GP9*)

### 3.3 Focus group with patients

The focus group was conducted to explore patients' perceptions of their current hypertension management, their interactions with HCPs, their views on interprofessional collaboration between pharmacists and GPs, and the key requirements for a potential pharmacy-based service for hypertension management. Four main themes were identified: 1) Perceived importance of a patient-centered co-care service, 2) experiences in pharmacies and future requirements, 3) experiences with GPs, and 4) communication among HCPs. Based on a single focus group with six participants, the findings provide exploratory insights into patient perspectives.

#### 3.3.1 Perceived importance of a patient-centered co-care service

Patients highlighted the importance of their personal perception of symptoms, as well as their individual needs, which can differ from guidelines and should be considered by HCPs in patient care. They questioned the standard blood pressure cut-off values and noted that medication may not always be necessary, even when blood pressure falls outside the defined target range. Additionally, patients highlighted the importance of holistic care, warning that a narrow focus on hypertension may overlook other key health concerns.

*«What does high blood pressure actually mean? Well, I know that most GPs (…) say that if your blood pressure is around 140, that is high and you definitely need medication. (…) Sure, 140. In theory, that is what it says in the books, but every person is different. For some people, 150 is OK, while for others, 140 is already far too high.» (P3)*

*«And then I was given so much blood pressure medication that my diabetes went even higher. But I didn't get any better. […] That's why I say the body is like a symbiosis.» (P6)*

#### 3.3.2 Experiences in pharmacies and future requirements

Patients' experiences with the pharmacy team, emphasizing the importance of trust, continuity, and confidential interactions were discussed. Some appreciated the convenience of having their blood pressure measured directly at the pharmacy, thereby avoiding a GP visit. They also noted that pharmacies could provide counseling of comparable quality to GPs. However, others did not perceive pharmacies as a medical authority, reported never visiting them, or considered pharmacists less competent than GPs.

«*The one who advised you. She took her time and, just like with the blood pressure earlier, she said, “Yes, this value can also be very individual.” But she says she is very satisfied. I should come back in six months, so I had a really good experience, and I don't necessarily have to go to the GP.*» *(P3)*

Participants emphasized the importance of having a designated point of contact in the pharmacy who is familiar with their medical history and current treatments to build trust. Some expressed dissatisfaction when their first contact was a pharmacy assistant instead of the pharmacist, who was often seen working out of sight. Moreover, participants mentioned the need for more privacy when discussing their health conditions in pharmacies, suggesting, like HCPs, that this kind of service should be conducted in a separate consultation room.

«*One word perhaps “contact person”. (…) If we had a contact person at the pharmacy, it would of course solve many of these problems. They would know my [medical] history, they would know what I take (…) then over time I would also have confidence.*» *(P5)*

#### 3.3.3 Experience with GPs

Participants reported mixed experiences with GPs, emphasizing the importance of communication and care consistency for a new co-care service. The most valued qualities in a GP were taking time to listen and referring patients to specialists for issues falling outside their area of expertise. Negative experiences included long waiting times, the perception that some GPs did not prioritize patient-centered care, and difficulty of finding care providers who were still accepting new patients. Additionally, a patient perceived self-dispensing GPs positively, as it avoided an additional visit to another healthcare professional.

« *I have a really good GP who took the time when he didn't know what to do and said, I can't get any further, I am sending you to a specialist*» *(P4)*

«*I am no longer satisfied because they [GP] no longer attend to the patient's needs.*» *(P4)*

Participants identified inconsistencies in blood pressure reference values among GPs and other HCPs as a challenge that contributed to mistrust toward some providers.

#### 3.3.4 Communication among HCPs

Participants discussed the challenges and opportunities related to sharing health information among HCPs in any interprofessional collaboration. They pointed out the burden of repeatedly communicating their health conditions and medication histories to multiple HCPs.

«*I had to tell my whole story over and over again and that's really tedious. It's very tedious.*» *(P3)*

Some participants shared positive experiences with tools that stored key patient health information, which could be accessed by various HCPs. Patients expressed interest in broader implementation of these tools.

«*(…) something very positive that I have experienced (…) in various departments [of a hospital]. I go and say to the women's clinic, for example, that I have this and that problem, and they say “Ah, they still have this and this and that”, they have a new tool and I don't have to explain everything I have to the GP (…)» (P4)*

However, concerns about data safety were raised. Participants feared that broad access to sensitive data by multiple stakeholders might compromise patient safety.

«*The problem is that the bigger these systems get, the more sensitive they become. And the more people have access to any data, the easier it is for a hacker to get in somewhere.*» *(P2)*

## 4 Discussion

This study aimed to identify key elements that may support the future development of a co-care service for chronic care management in patients living with hypertension, using the perspectives and experiences of pharmacists, GPs, and patients through a participatory qualitative approach. Interviews with HCPs provided important insights into the key structural components, challenges, and opportunities for an effective co-care service, as well as identifying some implementation barriers. In addition, the focus group with patients gave an overview of essential aspects to consider in this co-care service. These insights provide an initial glimpse into HCPs' and patients' views and should be interpreted as exploratory. HCPs expressed interest in developing such a service and agreed that involving pharmacists could enhance support for hypertension management. The participants discussed important structural aspects such as effective communication, interprofessional collaboration, mutual trust, clear role differentiation, and the Swiss political context.

Improving communication was identified as one of the main priorities by HCPs and patients. For both, implementing an EHR would improve communication through effective data sharing and reduce patients' burden of repeating the same health information to different HCPs. Yet, patients were concerned about data safety, which was also mentioned in a survey of 1963 Swiss residents, where administrative burden and usability concerns were also reported.^19^ Nevertheless, participants of the survey perceived an EHR to enhance access, efficiency, and quality of care. Similar benefits were experienced in British pharmacies, with an EHR with access to clinical records held by GPs, decision-support templates, and booking appointments.^20^ This EHR enhanced HCPs' relationship through communication, collaboration, and trust between pharmacies and GP practices. In line with this approach, an application is currently being tested in Switzerland as a pilot project, aiming to support digital information exchange and cardiovascular risk management between patients and HCPs.^21^ Such initiatives could be integrated into co-care services and contribute positively to improving communication and care coordination among HCPs, highlighting the importance of ensuring that EHRs are actively used in Switzerland.

For all participants, interprofessional collaboration and mutual trust were mentioned as being essential for an effective co-care service. Personal familiarity between HCPs and patients was seen as key to developing trust. Focus group participants emphasized the need for a designated contact person within the pharmacy. Interviewed HCPs highlighted the importance of locally defined collaboration, with pharmacists and GPs knowing one another personally to foster mutual trust. This principle has already been applied in a non-self-dispensation French-speaking region of Switzerland, through physician-pharmacist quality circles (PPQCs).^22^ PPQCs' involved at least one trained pharmacist and 3–10 local GPs, with the goal of promoting structured and continuous quality improvement and education, supported by local HCP networking to optimize prescribing practices. PPQC models could be used as basis to develop local HCP networks with a common language, thereby establishing mutual trust based on shared practice agreements. Such a co-care service could represent an initial step toward strengthening structured interprofessional collaboration and building sustainable local partnerships between pharmacists and GPs and support more coordinated hypertension management.

Another key element identified was clear role differentiation between GPs and pharmacists, which reflects stakeholder expectations and may help guide future development of a co-care service. Participating GPs and pharmacists were both interested in strengthening pharmacists' skills, a view shared by patients, who consider the quality of counseling provided in pharmacies to be comparable to that of GPs. While most HCPs recognized the potential for pharmacists to adjust medications in collaboration with prescribing GPs, they stressed that this would necessitate training beyond the current Swiss curriculum. In other countries such as England^23^ or Canada,^9^ pharmacists already adjust medication or have the authority to prescribe them (= prescribing pharmacists). In a Canadian randomized controlled trial,^23^ prescribing pharmacists significantly improved hypertension outcomes, achieving clinically and statistically significant reductions in systolic blood pressure at 6 months and higher rates of blood pressure control compared with usual care. However, clear practice procedures and guidelines must be established to support these expanded responsibilities. One possibility would be the use of the German traffic light guidelines,^10^ which were considered a useful baseline, to harmonize practice and establish clear triage rules and responsibilities within a local network of pharmacists and GPs. Additionally, Swiss pharmacists are familiar with the cardiovascular risk assessment service HerzCheck®,^13^ which also uses a traffic light color system to evaluate patient risk. To address concerns regarding overly strict blood pressure thresholds, the German traffic light guidelines, which pharmacists expressed willingness to continue using,^10^ could be adapted to the requirements of the co-care service. This could provide a standardized framework for risk assessment, patient referral, and collaborative management.

In Swiss cantons where GPs are allowed to dispense medications, the participants described political and regulatory context may limit opportunities to develop hypertension co-care service. Patients living in these cantons viewed self-dispensation positively, as it reduces the need for additional travel to obtain medication in a pharmacy. This may also result in less frequent contact with community pharmacies, potentially limiting familiarity with pharmacists and their roles. In addition, collaboration between GPs and pharmacists was described as limited, which was mentioned by participants as key element of the co-care service. These contextual differences across cantons should therefore be considered when planning the implementation of such a service in Switzerland. In this context, referral pathways could enable GPs to refer patients requiring additional information or support from pharmacists, thereby facilitating access to complementary care. Such co-care service was also perceived as a way to reduce the overall workload of GPs, which is particularly important in the context of a growing and ageing population^24^ and the current GP shortage.^25^ The latter was highlighted in a cross-sectional study, which estimated that 44% of GP full-time equivalents in Switzerland are expected to leave the workforce within the next 10 years.^25^

### 4.1 Strength and limitations

This study has several strengths and limitations. A key strength lies in the inclusion of multiple stakeholder perspectives, including pharmacists, GPs, and patients, allowing a comprehensive understanding of how a future co-care service could be structured in Swiss primary care settings. Participating HCPs were recruited from different Swiss regions, capturing linguistic, geographic, and regulatory contexts. A limitation was that participants were selected using a convenience and purposive sampling method, meaning that more engaged individuals, particularly HCPs with greater interest in new services and interprofessional collaboration, may have been more likely to participate. Similarly, it can be supposed that participating patients were better informed about their condition and its implications, since most were recruited through disease-specific patient organizations. In addition, included patients were only recruited from the German speaking part of Switzerland, thereby excluding perspectives from French- and Italian-speaking patients, which may limit the representativity of the findings across Switzerland. Additionally, hypertension was self-reported by patients, which was deemed appropriate for this qualitative exploratory design, as the study focused on patients' experiences within the Swiss healthcare pathway rather than clinical aspects of hypertension. HCPs responded based on a hypothetical co-care service rather than practical experience, which may have resulted in more favorable perspectives than would occur in real-world implementation. Overall, these factors may have contributed to a more positive perception of the proposed co-care service than might be observed in routine clinical practice and may limit the transferability to other HCPs. While HCPs and patients identified several key elements perceived as important for an effective co-care service, these findings do not necessarily indicate that all components are currently feasible within the Swiss healthcare system. Although existing local initiatives and national digitalization strategies provide a supportive context for some elements, implementation would depend on different factors, including local regulatory frameworks, reimbursement mechanisms, workforce capacity, inter-organizational workflows, and digital infrastructure. To reduce interpretation bias, data were independently coded and discussed by three pharmacist researchers (MC, AR, FM) until consensus was reached. All perspectives were considered equally to ensure a balanced representation of participant experiences in the analysis. Despite consensus-based discussions, researcher disciplinary backgrounds may have influenced interpretation, potentially emphasizing the perceived benefits of co-care services.

## 5 Conclusion

Based on participants' perspectives and experiences, this study explores key elements which may help guide the future development of a co-care service for patients with hypertension. Clear collaboration guidelines can help define HCPs' roles and responsibilities to ensure seamless care coordination and consistent patient information exchange. Additionally, patients' individual needs should be at the center of this service, and their preferences should be considered. The implementation of a national integrated and standardized EHR would facilitate communication and coordination among HCPs and patients. HCPs emphasized the potential benefits of this co-care model for patients, noting that integrating pharmacists could support GPs in managing workload, optimizing time, and improving care coordination. The service could be accepted for implementation where HCPs are already collaborating within an established and trusted network. The co-care service's foundation depends on a local way of communication and collaboration among HCPs, combined with a holistic patient-centered approach. A subsequent Phase 2 study will explore the facilitators and barriers as well as evaluate the feasibility of implementing this co-care service from the perspectives of HCPs and patients, using focus groups with HCPs and interviews with patients.

## CRediT authorship contribution statement

**Fanny Mulder:** Visualization, Project administration, Methodology, Investigation, Formal analysis, Conceptualization, Writing – original draft. **Marina Lopes Cardoso:** Methodology, Investigation, Formal analysis, Conceptualization, Writing – review & editing. **Alessia Romer:** Methodology, Investigation, Formal analysis, Writing – review & editing. **Kristie Rebecca Weir:** Visualization, Validation, Writing – review & editing. **Alice Panchaud:** Visualization, Validation, Supervision, Resources, Funding acquisition, Writing – review & editing. **Stephen Philip Jenkinson:** Visualization, Validation, Supervision, Investigation, Conceptualization, Writing – review & editing. **Karen Alexandra Maes:** Visualization, Validation, Supervision, Investigation, Conceptualization, Writing – review & editing.

## Funding

This research did not receive any specific grant from funding agencies in the public, commercial, or not-for-profit sectors.

## Declaration of competing interest

The authors declared no conflict of interest.

## Appendix A Supplementary data

COREQ (COnsolidated Criteria for Reporting Qualitative Research) guidelines and Topic Guides.COREQ (COnsolidated Criteria for Reporting Qualitative Research) guidelines and Topic Guides.
