# Supplementary material for: Exploration of a co-care service for chronic care management in patients with hypertension: A participatory qualitative study (Phase 1)
Source: Explor Res Clin Soc Pharm. 2026 Sep 13;24:100842. doi: 10.1016/j.rcsop.2026.100842 (PMC13627792; doi:10.1016/j.rcsop.2026.100842)
Supplement: Supplementary file 1 [file mmc1.docx]

Appendix A – COREQ (COnsolidated criteria for REporting Qualitative research) guidelines

| **No** | **Item** | **Guide questions/description** | **Our study** |
| --- | --- | --- | --- |
| **Domain 1: Research team and reflexivity** | | | |
| **Personal Characteristics** | | | |
| 1. | Interviewer/facilitator | Which author/s conducted the interview or focus group? | Methods, p.4 – Interviews with pharmacists and GPs: *MC, a novice interviewer, conducted all interviews (…)*  Methods, p.5 – Focus group with patients: *It was moderated by JL (…)* |
| 2. | Credentials | What were the researcher's credentials? *E.g. PhD, MD* | Title page of the manuscript:  **CRediT authorship contribution statement**  Fanny Mulder: Conceptualization, Formal analysis, Investigation, Methodology, Project administration, Visualization, Writing – original draft; Marina Lopes Cardoso: Conceptualization, Formal analysis, Investigation, Methodology, Writing – review and editing; Alessia Romer: Formal analysis, Investigation, Methodology, Writing – review and editing; Dr. Kristie Rebecca Weir: Validation, Visualization, Writing – review and editing; Prof. Dr. phil. Alice Panchaud: Funding acquisition, Resources, Supervision, Validation, Visualization, Writing – review and editing; Dr. sc. nat. Stephen Philip Jenkinson: Conceptualization, Investigation, Supervision, Validation, Visualization, Writing – review and editing; Dr. phil. II. Karen Alexandra Maes: Conceptualization, Investigation, Supervision, Validation, Visualization, Writing – review and editing |
| 3. | Occupation | What was their occupation at the time of the study? | Methods, p.4 – Interviews with pharmacists and GPs: *MC, a pharmacy master’s student, and refined with collaborative input from the research team. It was reviewed by FM, a pharmacist and PhD student, as well as KAM and SPJ, senior pharmacists with clinical and research expertise.*  Methods, p.5 – Focus group with patients: *It was moderated by JL, with a background in communication science and sociology. AR, a pharmacist and PhD student and AS, with qualifications in psychological and social sciences, served both as co-facilitators and workshop observers.* |
| 4. | Gender | Was the researcher male or female? | Not reported; gender can be inferred from authors’ names. |
| 5. | Experience and training | What experience or training did the researcher have? | Methods, p.4 – Interviews with pharmacists and GPs: *MC, a pharmacy master’s student, and refined with collaborative input from the research team. It was reviewed by FM, a pharmacist and PhD student, as well as KAM and SPJ, senior pharmacists with clinical and research expertise.*  Methods, p.5 – Focus group with patients: *It was moderated by JL, with a background in communication science and sociology. AR, a pharmacist and PhD student and AS, with qualifications in psychological and social sciences, served both as co-facilitators and workshop observers.*  Method, p.5 - *KW, an experienced qualitative researcher, supported the structuring of the analysis and critically reviewed the interpretation of participants’ perspectives.* |
| **Relationship with participants** | | | |
| 6. | Relationship established | Was a relationship established prior to study commencement? | Methods, p.4 - *No prior relationship was established between researchers and participants, and HCPs had no access to the interview questions or study aims, apart from the information in the invitation letter (see Appendix C – Invitation letter for a qualitative interview).* |
| 7. | Participant knowledge of the interviewer | What did the participants know about the researcher? e*.g. personal goals, reasons for doing the research* | Methods, p.4 - *No prior relationship was established between researchers and participants, and HCPs had no access to the interview questions or study aims, apart from the information in the invitation letter (see Appendix C – Invitation letter for a qualitative interview).* |
| 8. | Interviewer characteristics | What characteristics were reported about the interviewer/facilitator? e.g. *Bias, assumptions, reasons and interests in the research topic* | Methods, p.6 - *The qualitative study was conducted by a research team, with the analysis primarily performed by pharmacists (FM, MC, AR, SPJ, KAM) engaged in research promoting interprofessional c*  *ollaboration and the pharmacist’s role. KW, an experienced qualitative researcher, supported the structuring of the analysis and critically reviewed the interpretation of participants’ perspectives*. |
| **Domain 2: study design** | | | |
| **Theoretical framework** | | | |
| 9. | Methodological orientation and Theory | What methodological orientation was stated to underpin the study? *e.g. grounded theory, discourse analysis, ethnography, phenomenology, content analysis* | Methods, p.5 - *Three investigators (MC, AR, FM) conducted the step-by-step process of thematic analysis by Naeem et al. (18). MC conducted the analysis of all interviews and the focus group transcripts.* |
| **Participant selection** | | | |
| 10. | Sampling | How were participants selected? *e.g. purposive, convenience, consecutive, snowball* | Methods, p.3 – Interviews with pharmacists and GPs: *Participants were invited for an individual semi-structured interview via email using convenience and purposive sampling. Participating pharmacists were recruited through the Swiss Pharmacists Association pharmaSuisse, while GPs were recruited through the network of the Institute of Primary Care, University of Bern.*  Methods, p.4 – Focus group with patients: *For the patients’ focus group, a convenience sample of six patients were recruited from consumer groups including the Diabetes Club, the Heart Foundation Affected Council, and Diabetes Switzerland, and a recruitment agency.* |
| 11. | Method of approach | How were participants approached? e*.g. face-to-face, telephone, mail, email* | Methods, p.3 – Interviews with pharmacists and GPs: *Participants were invited for an individual semi-structured interview via email using convenience and purposive sampling.*  Methods, p.4 – Focus group with patients: *For the patients’ focus group, a convenience sample of six patients were recruited from consumer groups including the Diabetes Club, the Heart Foundation Affected Council, and Diabetes Switzerland, and a recruitment agency.* |
| 12. | Sample size | How many participants were in the study? | Results, p.6 – Interviews with pharmacists and GPs: *The HCPs interviewed included six pharmacists and six GPs*  Results, p.6 – Focus group with patients: *Six patients participated in the focus group* |
| 13. | Non-participation | How many people refused to participate or dropped out? Reasons? | Not applicable |
| **Setting** | | | |
| 14. | Setting of data collection | Where was the data collected? e*.g. home, clinic, workplace* | Methods, p.4 – Interviews with pharmacists and GPs: *The semi-structured interviews with pharmacists and GPs were conducted and recorded online via the platform Zoom® in April 2024.*  Methods, p.5 – Focus group with patients: *The focus group was led in person in March 2024 and took place in an external office in Bern, Switzerland.* |
| 15. | Presence of non-participants | Was anyone else present besides the participants and researchers? | Methods, p.4 – Interviews with pharmacists and GPs: *MC, a novice interviewer, conducted all interviews, with supervision provided by FM or SPJ during the first four interviews to ensure methodological rigor before MC proceeded independently.*  Methods, p.5 – Focus group with patients: *AR, a pharmacist and PhD student and AS, with qualifications in psychological and social sciences, served both as co-facilitators and workshop observers. MC also observed the focus group.* |
| 16. | Description of sample | What are the important characteristics of the sample? *e.g. demographic data, date* | Date:  Methods, p.4 – Interviews with pharmacists and GPs: *via the platform Zoom® in April 2024.* Methods, p.5 – Focus group with patients: *The focus group was led in person in March 2024 (…)*  Demographic data:  Results, p.6: *The HCPs interviewed included six pharmacists and six GPs (Table 1), with an even distribution of German- and French-speaking interviews. The sample included HCPs with under one year to 31 years of experience, with a median of 11.3 years. Most HCPs worked in regions where only pharmacists dispense medication, but other medication-dispensing models were also covered.*  *Six patients participated in the focus group (Table 2). All patient participants were German-speaking, and gender was evenly distributed. All participants self-reported a diagnosis of hypertension, and three had diabetes. The median age of patients was 62 years*. |
| **Data collection** | | | |
| 17. | Interview guide | Were questions, prompts, guides provided by the authors? Was it pilot tested? | Methods, p.3/4 – Interviews with pharmacists and GPs: *The interview topic guide was developed based on a literature review with a focus on the current management of hypertension in pharmacies and GP practices, participants’ views on how communication should occur, and their vision for a new hypothetical co-care service for managing hypertension (see Appendix B – HCPs’ interview topic guide).* / *A single pilot interview with a pharmacist led to minor adaptations of the interview topic guide, and its data was excluded from the analysis.*  Methods, p.5 – Focus group with patients: *No pilot was conducted prior to the focus group.* |
| 18. | Repeat interviews | Were repeat interviews carried out? If yes, how many? | Not relevant – No repeat interviews were conducted. |
| 19. | Audio/visual recording | Did the research use audio or visual recording to collect the data? | Methods, p.4 – Interviews with pharmacists and GPs: *The semi-structured interviews with pharmacists and GPs were conducted and recorded online via the platform Zoom® in April 2024.*  Methods, p.5 – Focus group with patients: *The focus group was led in person in March 2024 and took place in an external office in Bern, Switzerland., and was recorded using two independent audio recorders placed at opposite sides of the table to ensure comprehensive and safe audio capture.* |
| 20. | Field notes | Were field notes made during and/or after the interview or focus group? | No reported – No field notes were taken during or after the interviews or focus group. |
| 21. | Duration | What was the duration of the interviews or focus group? | Methods, p.4 – Interviews with pharmacists and GPs: *The interviews lasted between 28 to 50 minutes with a median interview duration of 34 minutes and 37 seconds.*  Methods, p.5 – Focus group with patients: *The focus group lasted 54 minutes.* |
| 22. | Data saturation | Was data saturation discussed? | Not applicable - As grounded theory methodology was not applied, data saturation was not formally assessed, in line with qualitative descriptive approaches. |
| 23. | Transcripts returned | Were transcripts returned to participants for comment and/or correction? | Methods, p.4 – Interviews with pharmacists and GPs: *A single pilot interview with a pharmacist led to minor adaptations of the interview topic guide, and its data was excluded from the analysis.*  Methods, p.5 – Focus group with patients: *The focus group integrated three thematic questions about co-care services for hypertension, based on the same literature review as the HCPs interview guide and development pathway, and no pilot was conducted prior to data collection.* |
| **Domain 3: analysis and findings** | | | |
| **Data analysis** | | | |
| 24. | Number of data coders | How many data coders coded the data? | Methods, p.5: *Three investigators (MC, AR, FM) conducted the step-by-step process of thematic analysis by Naeem et al. (18), using an inductive approach with themes derived from the data. MC conducted the analysis of all interviews and the focus group transcripts. AR independently analyzed the six German-language interviews, FM the French-language interviews, and both additionally contributed to the focus group’s transcript analysis.* |
| 25. | Description of the coding tree | Did authors provide a description of the coding tree? | Throughout the results: At the beginning of each theme, a brief description of the subthemes is provided to guide the reader through the structure of the results and to support interpretation of the main findings within each theme. |
| 26. | Derivation of themes | Were themes identified in advance or derived from the data? | Methods, p.5: *Three investigators (MC, AR, FM) conducted the step-by-step process of thematic analysis by Naeem et al. (18), using an inductive approach with themes derived from the data.* |
| 27. | Software | What software, if applicable, was used to manage the data? | Methods, p.5: *All transcripts were analyzed using MAXQDA® software (version 24.2.0).* |
| 28. | Participant checking | Did participants provide feedback on the findings? | Methods, p.5: *No transcripts were returned to participants for.* |
| **Reporting** | | | |
| 29. | Quotations presented | Were participant quotations presented to illustrate the themes / findings? Was each quotation identified? e*.g. participant number* | Yes – throughout the results |
| 30. | Data and findings consistent | Was there consistency between the data presented and the findings? | Yes – throughout the results |
| 31. | Clarity of major themes | Were major themes clearly presented in the findings? | Yes – throughout the results |
| 32. | Clarity of minor themes | Is there a description of diverse cases or discussion of minor themes? | Yes – throughout the results |

## Appendix B – HCPs’ interview topic guide

*The original interview topic guide was developed in German and French. It has been translated from French to English using DeepL Pro (DeepL GmbH, Cologne, Germany).*

1. **Preliminary questions:**

- How are patients who have already been diagnosed with hypertension by a general practitioner (GP) currently treated or managed in your pharmacy/medical practice?
  - Which internal or external professionals are involved in this process?

Imagine that there is a co-care approach in pharmacies and medical practices for patients who have already been diagnosed with hypertension by a GP. By “co-care”, we mean the shared management of a patient.

- How do you think this joint care should be structured or carried out?
  - *If only blood pressure measurement is mentioned*: Is there anything else that could be offered, or should the service be limited to blood pressure measurement in pharmacies?
- What would be the aim of this co-care?
- How do you envisage co-care ideally?
  - What would be the role of pharmacists and what would be the role of GPs?
  - Which other healthcare professionals should also be involved, and what tasks should they carry out?
  - How should the exchange of information take place?
- What would the framework conditions for such a co-care approach be?
  - *Support: premises, equipment/tools, required staff (e.g. time and costs), staff expertise*
- What are the obstacles to such a co-care approach?
- What are the opportunities for such a co-care approach?
- Do you consider such a service to be important?
  - Yes/No: please explain (strengths/weaknesses, benefits for patients, benefits for healthcare professionals)

2. **Main section (specific to blood pressure measurement):**

Now imagine a scenario in which the pharmacy measures blood pressure in patients who have already been diagnosed with hypertension, with the aim of monitoring their treatment. The pharmacy evaluates the results, provides recommendations and takes appropriate measures if necessary.

- What are your thoughts on this co-care approach for the patient, as well as on the collaboration between the pharmacy and the medical practice in this scenario?
- Do you see any added value for the patient?
- Do you see any added value in blood pressure measurement at the pharmacy compared to measurement at home?
- Do you see any added value for the treating medical practice?
- Do you see any added value for the pharmacy providing the follow-up care?
- What responsibilities should patient take on to support the success of their treatment?
- How do you assess your patients’ acceptance of such an interprofessional service?
- When would be the best time to start providing the service?
  - Guidance: *at the start of a new course of treatment / during treatment / after treatment adjustment?*
- How often should the service be offered in pharmacies?
- If a treatment adjustment is initiated by a GP, would blood pressure monitoring in a pharmacy be feasible?

In Germany, Schulz et al. have developed recommendations for pharmacies using a traffic-light system. Patients diagnosed with hypertension are encouraged to make an appointment with their GP within four weeks if their results fall within the red zone. If the results fall within the yellow zone, they are advised to inform their GP at their next medical appointment. If patients’ blood pressure results fall within the green zone, they are advised to continue monitoring or measuring their blood pressure regularly. To help you visualize this better, I’ll show you a corresponding image.

- *Show image:*

- Do you think such a classification is useful? Please explain
- What are your thoughts on the recommendations?
  - Can you envisage the pharmacist assessing and interpreting the measurement results?
  - Can you envisage the pharmacist adjusting the dose in the event of a ‘yellow’ classification, following consultation with the attending GP?
- How would you envisage communication between healthcare professionals in this context?
- What potential obstacles do you see to the successful implementation of this service?
- Would you be interested in taking part in such a co-care arrangement?

## Appendix C – Invitation letter for a qualitative interview

*The original invitation letter was developed in German and French. The invitation letter has been translated from French to English using DeepL Pro (DeepL GmbH, Cologne, Germany).*

**Invitation to a Qualitative Study**

My name is Marina Lopes Cardoso, and I am currently in my first year of the Master's degree program in Pharmacy at the University of Bern. During this semester, I am working on my master’s thesis at the Institute of Primary Health Care (BIHAM), under the supervision of Fanny Mulder and Dr. Stephen Jenkinson. The aim of my thesis is to take a first step toward the development of an interprofessional service for patients with hypertension.

I would like to invite you to participate in a qualitative study on the co-management of patients with hypertension in medical practices and pharmacies. Your opinion on this topic is extremely valuable, and I would be very grateful if you would agree to take part in this interview. Your contact details were provided to me by my supervisors, Dr. Stephen Jenkinson and Fanny Mulder.

The interview is expected to last approximately one hour and will include a series of questions regarding pharmacy services for patients with hypertension. This is a qualitative study during which I will ask you open-ended questions, and you will be free to respond as you wish. The conversation will be recorded, and the data will be anonymized. The aim is to gather the opinions and experiences of various healthcare professionals in order to gain a deeper understanding of the topic. Based on the responses obtained, a first draft of an interprofessional service will be developed.

The interviews are scheduled to take place during the month of April. The exact date and location will be agreed upon together in advance.

If you have any further questions or require additional information, please do not hesitate to contact me. Your participation and perspective would be greatly appreciated to the success of my master's thesis.

Thank you in advance for your support! I look forward to hearing from you.

Kind regards,

Study Team

## Appendix D – Patient interview topic guide

*The original patients interview topic guide was developed in German and French. The patients interview topic guide has been translated from French to English DeepL Pro (DeepL GmbH, Cologne, Germany)*

**Scenario**: You visit your GP regularly to have your high blood pressure checked. Imagine that you also had the option of receiving a service related to your high blood pressure at the pharmacy. In this case, your pharmacist would work in collaboration with your GP.

**1. General:**
   What type of counselling or service could you imagine receiving at a pharmacy?
**2. Interprofessional collaboration:**
   What has been your experience regarding communication between your pharmacist and GP during your treatment? Are there any positive or negative aspects, and is there anything that needs improving?
**3. Specific:**
   What would you think about having your blood pressure checked at the pharmacy every six months? Please explain your thoughts.
